# A Fused Maxillary Central Incisor and Its Multidisciplinary Treatment: An 18-Year Follow-Up

**DOI:** 10.1155/2014/503478

**Published:** 2014-02-11

**Authors:** Lluís Brunet-Llobet, Jaume Miranda-Rius, Eduard Lahor-Soler, Abel Cahuana

**Affiliations:** ^1^Pediatric Dental Unit, Servei d'Odontologia, Hospital Universitari Sant Joan de Déu. UB, Esplugues de Llobregat, 08950 Barcelona, Spain; ^2^Periodontics Unit, Departament d'Odontostomatologia, Universitat de Barcelona, L'Hospitalet de Llobregat, 08907 Barcelona, Spain; ^3^Endodontics Unit, Departament d'Odontostomatologia, Universitat de Barcelona, L'Hospitalet de Llobregat, 08907 Barcelona, Spain

## Abstract

Fused teeth may cause aesthetic, spacing, periodontal, eruption, and caries problems. The present case report describes a 7-year-old boy patient with a chief complaint of unerupted maxillary incisor. Radiographic examination indicated a fused tooth which had two fused roots but two independent root canals. A complex management of a fused tooth is really difficult to standardize. In this case an orthodontic, endodontic, and surgical treatment (intentional replantation) allowed the tooth to be retained until 18 years following intervention. Maintenance of the root and alveolar bone in young adults at least until full skeletal maturation should be the main treatment objective.

## 1. Introduction

Dentition development is a very complex process either in the primary or in the permanent tooth. Some authors have proposed a model of genetic network regulation in tooth formation; it seems obvious that most human congenital malformations and dental defects are caused by mutations in developmental regulatory genes. There would be a molecular basis of dental defects [1].

Fusion and gemination are considered abnormalities in tooth development. It is often difficult to differentiate between gemination and fusion and it was common to refer to these anomalies as “double teeth,” “double formations,” “joined teeth,” “fused teeth” or “dental twinning” [2–5].

In gemination the subdivision of the tooth bud is incomplete, giving rise to two dental units, the width of which in the mesiodistal dimension can be twice the dimensions of a single dental unit usually sharing a single root, pulp chamber and root canal. This bifid tooth is considered as a single tooth [6, 7].

By contrast, in fusion the originally separate tooth buds unite at the crown level (enamel) or at the crown and root levels (enamel and dentine), yielding a single large tooth during the odontogenesis, when the crown is not yet mineralized [8, 9].

Both anomalies occur more frequently in the primary dentition, particularly in the canine-incisor region, involving maxillary central and lateral incisors and mandibular lateral incisors and canines [2]. The incidence of unilateral occurrence is estimated in the literature to be 0.5% in the deciduous and 0.1% in the permanent dentition. There seems to be an overall lower incidence of double teeth in Caucasians than in Asians [10].

The aetiology of fusion is still unknown, but the influence of pressure or physical forces producing close contact between two developing teeth was reported as a possible cause. Gemination can be interpreted as an attempt of a supernumerary tooth to form [3]. Others believe that the basis of both anomalies is the persistence of dental lamina between two or more buds [11]. Genetic predisposition and racial traits were also reported as contributing factors [1].

This case report describes the treatment of a fused maxillary central incisor with a talon cusp using a multidisciplinary approach to manage and restore function and aesthetic appearance.

## 2. Case Report

**Figure 1:**
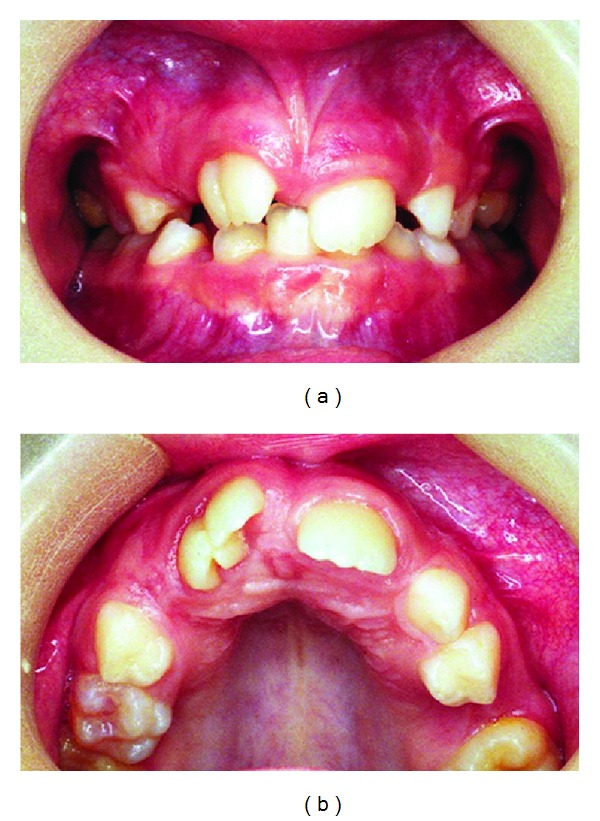
Clinical images of the fused right maxillary central incisor in eruption. Notice its broad crown with a talon cusp.

**Figure 2:**
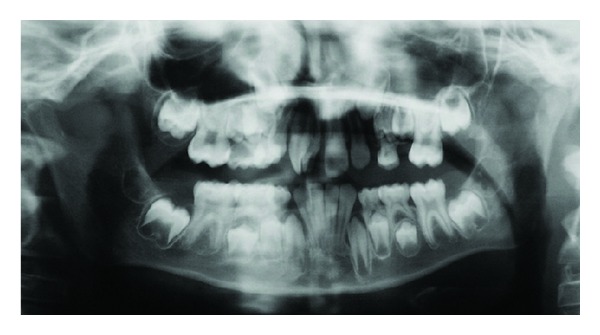
Panoramic radiography. Notice the fused tooth with two root canals.

In 1995, a 7-year-old boy was referred to Sant Joan de Déu Hospital complaining of unerupted maxillary right central incisor -tooth 11-. There was no significant past medical history nor family history of dental anomalies. The patient was in the first phase of mixed dentition stage. After radiographic examination, the preliminary diagnosis was a supernumerary tooth. Some months later, the incisor was present in the arch after a spontaneous eruption and the total number of teeth was normal. However, morphologically this central incisor showed macrodontia as a result of a fusion with a supernumerary tooth with a talon cusp (Figure 1). At that moment periapical and panoramic radiographic examination confirmed the diagnostic of a fused maxillary central incisor which had two fused roots but two independent root canal and two pulp chambers (Figure 2).

The molar relationship was a half unit Class II bilaterally. Moreover left side presented a severe osteodental discrepancy which made difficult the right lateral incisor eruption -tooth 12-.

At the age of 10, the treatment plan was explained to the patient and his family. The main aim was to reduce mesiodistal tooth size—hemisection—in order to allow lateral incisor eruption. As there was a high risk of pulp exposure during odontosection, due to its fused roots, previously root canal treatment was performed.

**Figure 3:**
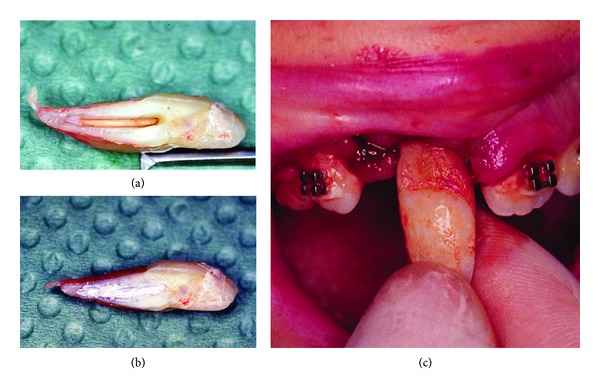
Intraoperative images. (a) After extraction, hemisection was performed. (b) Gutta-percha exposure was filled with silver amalgam. (c) The tooth's remaining portion was then replanted into the socket.

Under general anaesthesia a full-thickness buccal flap was reflected. After examining the outline and the position of the roots, the fused tooth was extracted and separated using a high-speed bur with water spray longitudinally throughout the root conjunction line. During this process gutta-percha was exposed and that moment it was decided to cover the root canal material with silver amalgam to avoid any filtration. The tooth's remaining portion was replanted into the socket and splinted to adjacent teeth with an orthodontic appliance. No orthodontic force was applied to it for 30 days (Figure 3).

**Figure 4:**
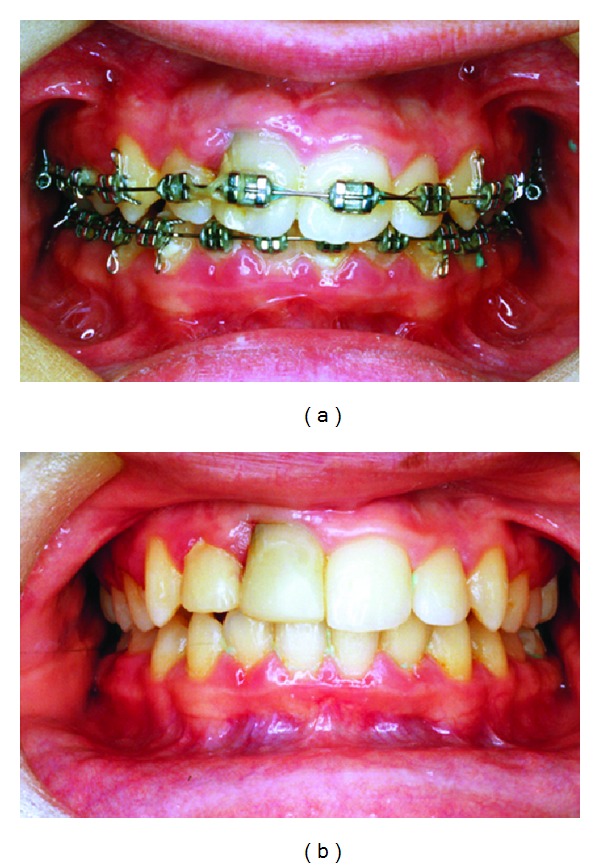
Clinical images. (a) Orthodontic treatment for Class II malocclusion; (b) dental cosmetic treatment of the right maxillary lateral and central incisors.

At the age of 13 orthodontic treatment for Class II malocclusion was initiated using an extraoral headgear and multibrackets (24 months). At the end of orthodontics a dental cosmetic treatment of these central and lateral incisors was indicated (Figure 4). At the age of 17 this patient was discharged from our hospital Paediatric Dental Unit with a satisfactory result.

**Figure 5:**
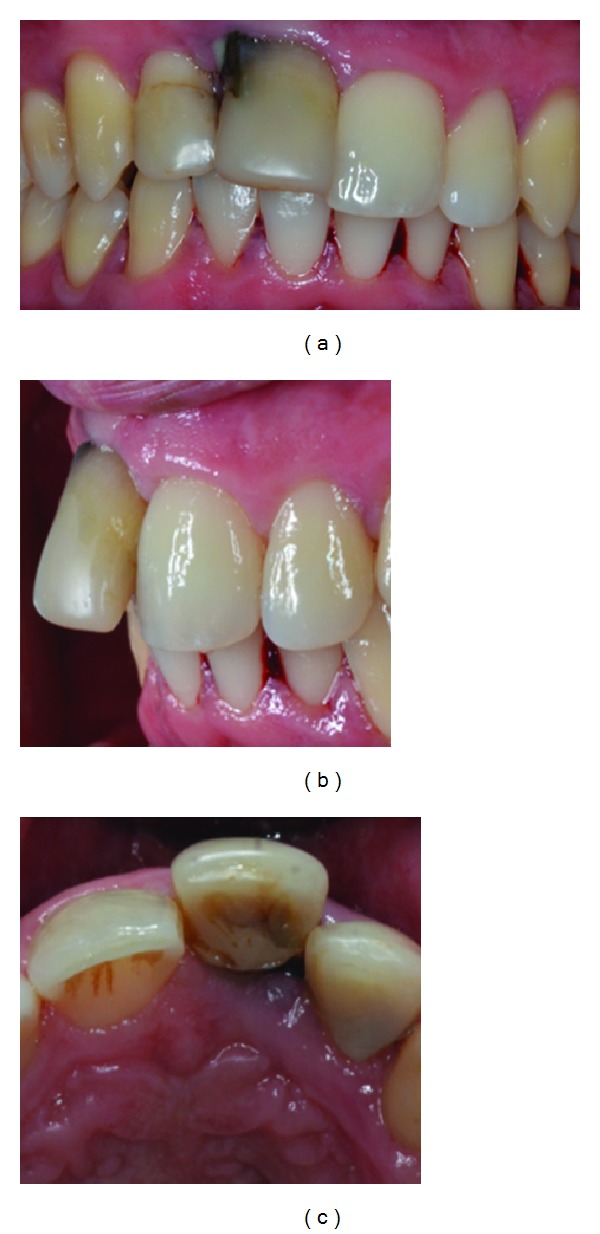
Clinical images after 18 years since the diagnosis. Notice a progressive pathological migration in buccal direction.

Eleven years later, at the age of 28, the patient was attended at Dental Hospital in the University of Barcelona and his complain was a progressive pathological migration of the right central incisor in buccal direction. Clinical exploration confirmed the buccal migration, a grey discoloration, an incremented probing depth (11 mm) at the interproximal area with right lateral incisor, some pus exudation from this periodontal pocket, and no dental vitality of this lateral incisor (Figure 5). However gingival margin remained at the same level of adjacent teeth.

**Figure 6:**
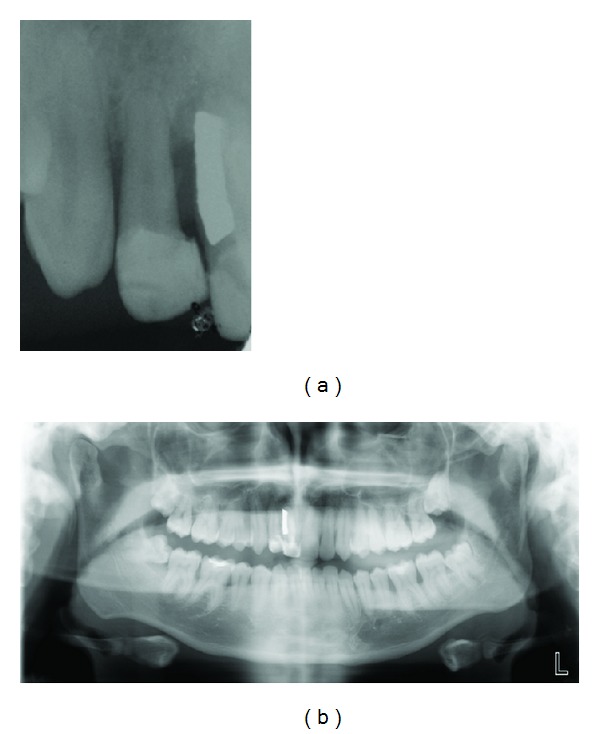
Periapical and panoramic radiographs showed a severe vertical bone defect.

Periapical radiograph showcased an apical image involving lateral right incisor and a severe vertical bone defect between right central and right lateral incisor, but the alveolar mesial bone level was preserved (Figure 6). Oral Surgeon indicated endodontic treatment of the lateral incisor and the restitution of this central incisor by an unitary dental implant with previous bone regeneration.

## 3. Discussion

Just as important is the identification of the possible anatomic variations and different anomalies present in all tooth groups. One of these anomalies causing major difficulties for diagnosis and treatment is tooth fusion, which may be commonly confused with tooth gemination.

Although the aetiology of these anomalies is still unknown, it is believed that some physical force or pressure/trauma causes the contact of developing teeth. Genetic predisposition and racial traits were also reported as contributing factors [8, 12].

Clinically a fusion of a regular tooth and a supernumerary tooth may result in crowding, protrusion, or the impactation of an adjacent tooth owing to insufficient arch length [13]. Several complications may occur: caries in the groove between the fused crowns leading to endodontic treatment, if not treated; tooth impactation, diastemas, and aesthetic and periodontal problems, which often demand a multidisciplinary treatment [14].

*Dens evaginatus* is the malformation of a tooth characterized by the presence of an accessory cusp. Talon cusp refers to the same condition but it is manifested on anterior teeth [8, 15, 16]. In the literature there are a few reports observing the anomalies of dental fusion and dens evaginatus in the same tooth, like in our case, which is considered a rarity [8, 17].

Clearly, a careful clinical and radiographic examination is beneficial for optimal treatment planning. However, Computerized tomography (CT) has the potential to visualize the topography of the root canals, offers new perspectives for dental imaging for special clinical cases, and may confirm the exact path of the root canal [17–19].

Several treatment methods are described in the literature with respect to the different types and morphologic variations of fused teeth [8]. Case reports have described the multidisciplinary treatment of fused permanent teeth, comprising extraction, endodontic treatment, tooth mesiodistal dimension reduction followed by orthodontic treatment, tooth hemisection, and intentional replantation [2, 20]. In this case most of these procedures were performed.

The treatment of fused teeth may be complex and contain various treatment protocols [21–24]. The reduction of mesiodistal dimensions with intraoral or extraoral hemisection of the tooth or the root with intentional dental replantation was illustrated by numerous case reports [23, 25]. Intentional replantation is a technique aimed mainly at the resolution of endodontic pathosis impossible to treat by conventional orthograde endodontic therapy, and with contraindications for apical surgery. Other reasons for intentional dental replantation, described in the literature, are treatment of extrusive dislocation and periodontally compromised teeth [2]. This method also proves useful when maintenance of the alveolar bone is necessary for prosthetic and implant treatment [26]. In our case we need to do a hemisection of the crown and the root (intentional replantation) because there was crowding, impactation, and difficult dental eruption of adjacent teeth.

The success of intentional replantation, estimated as the tooth retention rate, is reported to be above 67% and up to 93% and seems to be linked to three factors: previously existing endodontics pathosis and chronic infection, the length of extraoral treatment, and type of splinting. The time from extraction to replantation and the preservation and handling methods of the tooth are probably crucial for maintaining vitality of the periodontal ligament [27].

In our case, with the aim to avoid cracks on root and apical surface after tooth hemisection, the root planning was done with multifluted burs. At the end of this procedure, root must present a plane and smooth aspect, without degrees or irregularities that could act as irritants and stimulated root dentin resorption during periapical healing. Currently, the root-end cavity would be filled with Mineral Trioxide Aggregate (MTA). MTA is a biocompatible dental material which allows the proliferation of periodontal ligament cells, promotes an adequate sealing, and reduces apical leakage [28–30]. However in 1995, this new dental material was unknown and the best material for periapical surgery was silver amalgam. That is the reason why, in our case, we used the amalgam to seal the longitudinal radicular gap.

Maintenance of the tooth and the alveolar bone is nevertheless crucial in growing patients: both from the psychological point of view as well as for hard and soft tissue maintenance, which, in case of an unsuccessful replantation because of resorption and/or root ankylosis, may facilitate implant therapy in adulthood [2, 26]. In the case presented, at the 18-year follow-up (1995–2013), there was a deep periodontal pocket between tooth 11 and tooth 12 around the silver amalgam area, with an infrabony defect, without clinico-radiological evidence of root resorption and/or ankylosis. A bone regeneration process will be necessary to allow a satisfactory implant surgical procedure. In case that this patient had lost the fused tooth at its earlier growing stages would have been many chances of a serious localized alveolar atrophy.

## 4. Conclusion

This case report provides, after 18 years, relevant information to the clinicians because it has a considerable follow-up. Additionally it illustrates that the treatment of a double tooth may be really difficult to standardize. Preservation of teeth during the age of development, even with uncertain prognosis, appears crucial for maintenance of the anatomy of the alveolar process for eventual implant therapy in adulthood.

## References

[1] Thesleff I (2000). Genetic basis of tooth development and dental defects. *Acta Odontologica Scandinavica*.

[2] Sivolella S, Bressan E, Mirabal V, Stellini E, Berengo M (2008). Extraoral endodontic treatment, odontotomy and intentional replantation of a double maxillary lateral permanent incisor: case report and 6-year follow-up. *International Endodontic Journal*.

[3] Duncan WK, Helpin ML (1987). Bilateral fusion and gemination: a literature analysis and case report. *Oral Surgery, Oral Medicine, Oral Pathology, Oral Radiology and Endodontology*.

[4] Brook AH, Winter GB (1970). Double teeth. A retrospective study of “geminated” and “fused” teeth in children. *British Dental Journal*.

[5] Yuen SW, Chan JC, Wei SH (1987). Double primary teeth and their relationship with the permanent successors: a radiographic study of 376 cases. *Pediatric Dentistry*.

[6] Rada RE (1991). Perio-prosthetic rehabilitation of a geminated central incisor. *Practical Periodontics and Aesthetic Dentistry*.

[7] Aryanpour S, Bercy P, van Nieuwenhuysen J-P (2002). Endodontic and periodontal treatments of a geminated mandibular first premolar. *International Endodontic Journal*.

[8] Ozden B, Gunduz K, Ozer S, Oz A, Ozden FO (2012). The multidisciplinary management of a fused maxillary central incisor with a talon cusp. *Australian Dental Journal*.

[9] Schuurs AHB, van Loveren C (2000). Double teeth: review of the literature. *Journal of Dentistry for Children*.

[10] Tasa GL, Lukacs JR (2001). The prevalence and expression of primary double teeth in western India. *Journal of Dentistry for Children*.

[11] Surmont PA, Martens LC, de Craene LG (1988). A complete fusion in the primary human dentition: a histological approach. *Journal of Dentistry for Children*.

[12] Cetinbas T, Halil S, Akcam MO, Sari S, Cetiner S (2007). Hemisection of a fused tooth. *Oral Surgery, Oral Medicine, Oral Pathology, Oral Radiology and Endodontology*.

[13] Melnik AK (1993). Orthodontic movement of a supplemental maxillary incisor through the midpalatal suture area. *The American Journal of Orthodontics and Dentofacial Orthopedics*.

[14] Atasu M, Cimilli H (2000). Fusion of the permanent maxillary right incisor to a supernumerary tooth in association with a gemination of permanent maxillary left central incisor: a dental, genetic and dermatoglyphic study. *Journal of Clinical Pediatric Dentistry*.

[15] Al-Omari MAO, Hattab FN, Darwazeh AMG, Dummer PMH (1999). Clinical problems associated with unusual cases of talon cusp. *International Endodontic Journal*.

[16] Hattab FN, Yassin OM, al-Nimri KS (1995). Talon cusp—clinical significance and management: case reports. *Quintessence International*.

[17] Mupparapu M, Singer SR, Goodchild JH (2004). Dens evaginatus and dens invaginatus in a maxillary lateral incisor: report of a rare occurrence and review of literature. *Australian Dental Journal*.

[18] Weglarz WP, Tanasiewicz M, Kupka T, Skórka T, Sułek Z, Jasiński A (2004). 3D MR imaging of dental cavities—an in vitro study. *Solid State Nuclear Magnetic Resonance*.

[19] Baratto Filho F, Zaitter S, Haragushiku GA, de Campos EA, Abuabara A, Correr GM (2009). Analysis of the internal anatomy of maxillary first molars by using different methods. *Journal of Endodontics*.

[20] Mancuso A (2003). The treatment of fusion and supernumerary maxillary central incisors: a case report. *General Dentistry*.

[21] Tuna EB, Yildirim M, Seymen F, Gencay K, Ozgen M (2009). Fused teeth: a review of the treatment options. *Journal of Dentistry for Children*.

[22] Malcic Prpic-Mehicic G (2005). Conservative treatment of fused teeth in permanent dentition. *Acta Stomatologica Croatica*.

[23] Olivan-Rosas G, López-Jiménez J, Jiménez-Prats MJ, Piqueras-Hernández M (2004). Considerations and differences in the treatment of a fused tooth. *Medicina Oral*.

[24] Tsukiboshi M (2001). *Autotransplantation of Teeth*.

[25] Tsurumachi T, Kuno T (2003). Endodontic and orthodontic treatment of a cross-bite fused maxillary lateral incisor. *International Endodontic Journal*.

[26] Schwartz-Arad D, Levin L, Ashkenazi M (2004). Treatment options of untreatable traumatized anterior maxillary teeth for future use of dental implantation. *Implant Dentistry*.

[27] Andreasen JO, Borum MK, Jacobsen HL, Andreasen FM (1995). Replantation of 400 avulsed permanent incisors. Part 1. Diagnosis of healing complications. *Endodontics and Dental Traumatology*.

[28] Gerhardt OM, Rockenbach BMC, Wehmeyer FP, da Cunha Filho JJ, Puricelli E (2010). Scanning electron microscopy resection with burs and lasers. *Chirurg*.

[29] Samara A, Sarri Y, Stravopodis D, Tzanetakis GN, Kontakiotis EG, Anastasiadou E (2011). A comparative study of the effects of three root-end filling materials on proliferation and adherence of human periodontal ligament fibroblasts. *Journal of Endodontics*.

[30] Fernández YSA, Leco BMI, Martínez GJM (2008). Metaanalysis of filler materials in periapical surgery. *Medicina Oral*.

